# Clinical Utility of Immature Platelet Fraction (IPF) as a Biomarker in the Diagnosis of Acute Coronary Syndrome (ACS)

**DOI:** 10.7759/cureus.81406

**Published:** 2025-03-29

**Authors:** Vinayak K Sarma, Renoy A Henry, Hisham Ahamed, Sanjeev Mathew George

**Affiliations:** 1 Internal Medicine, Amrita Institute of Medical Sciences and Research Centre, Kochi, IND; 2 Cardiology, Amrita Institute of Medical Sciences and Research Centre, Kochi, IND

**Keywords:** acute coronary syndrome, immature platelet fraction, mean platelet volume (mpv), non-st segment elevation acute coronary syndrome, st-segment elevation myocardial infarction (stemi)

## Abstract

Introduction

Acute coronary syndrome (ACS) is a critical medical emergency requiring prompt diagnosis and treatment to prevent severe complications, including death, and the underlying pathology of ACS involves the rupture or erosion of an atheromatous plaque within the coronary arteries. Platelets get consumed in an atherosclerotic blood vessel (artery) soon after the rupture of atherosclerotic plaque, which can result in the release of larger immature platelets from bone marrow. The primary objective of this study was to determine the clinical utility of immature platelet fraction (IPF) as a biomarker in the diagnosis of ACS. As secondary objectives, we tried to determine the association between mean platelet volume (MPV) and IPF in the diagnosis of ACS and to determine the role of IPF in differentiating single vessel disease (SVD), double vessel disease (DVD), and triple vessel disease (TVD).

Materials and methods

A pilot study was conducted with 15 samples, and the sensitivity of IPF (90%) to diagnose ACS was obtained with a 95% confidence interval and 10% allowable error; the overall minimum sample size came to 53 (including a minimum 35 ACS). Fifty-four patients with chest pain, including patients who presented to the emergency room (ER) and who got admitted in the ward and critical care unit (CCU), satisfying the inclusion criteria, were included in the study. Statistical analysis was done using the IBM SPSS Statistics for Windows, Version 20 (IBM Corp., Armonk, NY). The results are given in mean ± SD for all the continuous variables and in frequency (percentage) for categorical variables. The normality of the data was checked by the Kolmogorov-Smirnov Z test. The receiver operating characteristic (ROC) curve was applied to find an ideal cut-off of IPF for the diagnosis of ACS with respect to ECG and cardiac enzymes. Diagnostic measures such as sensitivity and specificity were applied. To test the statistical significance of the difference in the proportion of IPF and MPV with ACS, the Chi-square test was used, and the same test was used to differentiate SVD, DVD, and TVD using IPF with respect to coronary angiogram (CAG). Pearson’s correlation coefficient was applied to find the correlation of IPF with MPV and platelet count, and its statistical significance was checked by linear reg t test.

Results

Among the 54 participants, 38 (70.4%) were diagnosed with ACS, while 16 (29.6%) were found to have non-cardiac chest pain. The ROC curve was plotted, and a cut-off value of 1.7250% was determined for IPF with a sensitivity of 94.7% and a specificity of 93.7%. The area under the ROC curve (AUC) was 0.984, with a standard error of 0.014 (p < 0.001). There was a strong positive correlation between IPF and MPV (r = 0.731, p < 0.001) in ACS patients.

Conclusion

Our study demonstrates that IPF and MPV are valuable biomarkers for the diagnosis of ACS.

## Introduction

Acute coronary syndrome (ACS) is defined as acute symptoms of the disease that arise from the rupture of atheroma plaque within the coronary blood vessels. It accounts for 85% of occurrences of coronary heart disease, affecting 17.9 million individuals globally. This is one among the serious cardiovascular events because it raises hospital admission and death rates [1]. It is a type of heart disease that is responsible for over one-third of total deaths happening in people above 35 years of age [2]. The three types of ACS include non-ST-segment elevation myocardial infarction (NSTEMI), ST-elevation myocardial infarction (STEMI), and unstable angina (UA). Myocardial infarction (MI) is another term for the two former disorders. Biochemical marker levels for myocardial necrosis in MI are elevated, while there is no elevation in UA [3]. 10% to 15% of ACS patients are misdiagnosed because, at the time of their initial examination in emergency rooms, their troponin I level and electrocardiogram (ECG) features are normal [4].

The American College of Cardiology Foundation, the American Heart Association, the World Heart Federation, and the European Society of Cardiology all state that cardiac troponin-T (cardiac TnT) and cardiac troponin-I (cardiac TnI) are the biomarkers that are currently used for the diagnosis of ACS [5]. Heart biomarkers have limited diagnostic value two to four hours before the disease onset sets in, when patients already have little and non-specific symptoms since their levels only increase three to five hours after the myocardial infarction has begun [6]. Notably, initial troponin I (initial TnI) levels do not allow for the diagnosis of 40-60% of ACS [7].

The blood circulation comes into contact with the thrombogenic substance found inside atherosclerotic plaque. Coronary artery blockage will result from platelet activation, the start of the coagulation cascade, and the creation of a mural thrombus. STEMI may be caused by a complete coronary artery blockage. Conversely, partial atherothrombosis results when there is an abrupt breakdown of plaque of atherosclerosis inside the coronaries, which in turn causes ACS, both UA and acute myocardial infarction (AMI) [8]. UA, represented by a partial blockage of the coronary artery, results in decreased flow of blood, whereas AMI is defined as atheromatous plaque totally blocking the coronary artery that in turn results in the necrosis of cardiomyocytes [4]. Platelets are essential for the development of thrombus and atherosclerotic plaque. Following the rupture of the atherosclerotic plaque, platelets participate in the production of thrombus after the rupture of atherosclerotic plaque. Consuming platelets in an atherosclerotic blood artery can cause the bone marrow to release more massive platelets. Because larger platelets express more glycoprotein IIb/IIIa and contain more prothrombotic components such thromboxane A2, they are metabolically and enzymatically more active [9]. Following the rupture or erosion of atherosclerotic plaques, platelet activation and recruitment lead to thrombus development [9]. Immature platelets can be released from the bone marrow.

The immature platelet fraction (IPF) can be defined as the absolute immature platelets counts (AIPC), which is the number of immature platelets present in unit volume (% IPF × platelet counts). The IPF is usually reported as the IPF percentage. Mean platelet volume (MPV) during a routine complete blood count examination correlates positively with platelet activity in addition to reflecting the size of the platelets. An increase in the volume of platelets is associated with enhanced adhesion surface molecule expression, thromboxane A2 production, β-thromboglobulin release, and platelet aggregation ability [10]. It is interesting to note that compared to healthy people, patients with diabetes, hypertension, high cholesterol, obesity, smoking habits, or other cardiovascular disease risk factors have greater MPV [11-13]. This suggests that MPV is a risk factor for cardiovascular disease in patients and could be a useful biomarker for the diagnosis of ACS. During the course of atherosclerosis, platelets are involved in both the breakdown of the endothelium and the plaque rupture. The interaction between platelets and the endothelium lining arteries results in increased platelet activation, which lowers the half-life and increases platelet turnover, ultimately affecting the MPV and IPF [14].

Aim and primary objective

The aim and primary objective of our study was to determine the significance of IPF as a biomarker in the diagnosis of ACS.

Secondary objectives

Additionally, we considered looking into the association between MPV and IPF in the diagnosis of ACS, the role of IPF in differentiating single vessel disease (SVD), double vessel disease (DVD), and triple vessel disease (TVD). The patients presenting to the emergency department with acute chest discomfort were evaluated in the study. The MPV and IPF of the patients who had a possibility of having ACS were examined, also connections among the occurrence of ACS and MPV or IPF were analyzed.

## Materials and methods

This was a single-center, prospective, cross-sectional study and was carried out over 12 months from May 2023 to May 2024 in the Department of General Medicine/Cardiology, Amrita Institute of Medical Sciences and Research Centre, Kochi, Kerala, India. The Ethics Committee of Amrita School of Medicine issued approval ECASM-AIMS-2023-207, dated April 11, 2023. Study subjects were patients presenting to the ER and patients admitted in the ward with acute chest pain. The study participants were recruited after counseling regarding the study and obtaining their fully informed consent. IPF and MPV were measured. Patients were followed up, and the results were analyzed statistically for the significance of the findings from the study.

Based on the sensitivity of IPF (90%) to diagnose ACS obtained from the pilot study conducted at Amrita Institute of Medical Sciences and Research Centre in 15 samples and with 95% confidence and 10% allowable error, the minimum number of ACS was 35. The prevalence rate of ACS was 66.66%. So, the overall minimum sample size comes to 53 (including a minimum of 35 ACS).

Inclusion criteria

Patients presenting to the emergency room (ER) and patients admitted in the ward with acute chest pain in our hospital.

Exclusion criteria

Exclusion criteria include patients with age <18 years, severe hepatic and/or renal disease, myeloproliferative disorders, malignancy, immune thrombocytopenic purpura (ITP), chest pain related to trauma, and those taking oral anticoagulant medications or previously diagnosed as ACS in eight weeks and treated accordingly.

Statistical analysis

Statistical analysis was done using the IBM SPSS Statistics for Windows, Version 20 (IBM Corp., Armonk, NY). The results are given in mean ± SD for all the continuous variables and in frequency (percentage) for categorical variables. The normality of the data was checked by the Kolmogorov-Smirnov Z test.

The receiver operating characteristic (ROC) curve was applied to find an ideal cut-off of IPF for the diagnosis of ACS with respect to ECG and cardiac enzymes. Diagnostic measures such as sensitivity and specificity were applied. To test the statistical significance of the difference in the proportion of IPF and MPV with ACS, the Chi-square test was used, and the same test was used to differentiate SVD, DVD, and TVD using IPF with respect to coronary angiogram (CAG). Pearson’s correlation coefficient was applied to find the correlation of IPF with MPV and platelet count, and its statistical significance was checked by linear reg t test. A p-value of < 0.05 was considered statistically significant. All tests of statistical significance were two-tailed.

## Results

During the study period of 12 months, a total of 54 patients with chest pain (including patients who attended the ER and those who were admitted in the ward) satisfying the inclusion criteria were included in the study. Thirty-eight (70.4%) patients were diagnosed with ACS, and 16 (29.6%) patients had non-cardiac chest pain. Among the 38 ACS patients, 11 (20.4%) patients were diagnosed with STEMI, 25 (46.3%) patients were diagnosed with NSTEMI, and two (3.7%) patients were diagnosed with UA.

Thirty-eight patients who were diagnosed with ACS have a mean age of 68.34 years (SD = 11.530), while 16 patients who were diagnosed with non-cardiac chest pain have a mean age of 64.06 years (SD = 15.185) (Table 1). The standard error of the mean is 1.870 for ACS patients and 3.796 for patients with non-cardiac chest pain.

**Table 1:** Age distribution of patients with ACS and non-cardiac chest pain. ACS, acute coronary syndrome; IPF, immature platelet fraction; MPV, mean platelet volume. Pearson’s correlation coefficient was applied to find the correlation of IPF with MPV and platelet count, and its statistical significance was checked by linear reg T test. A p-value of < 0.05 was considered statistically significant.

ACS/non-cardiac chest pain	Frequency	Mean age	Standard deviation	p-value
ACS	38	68.34	11.530	0.263
Non-cardiac	16	64.06	15.185	

Out of 54 patients who were enrolled in the study, 37 (68.5%) patients were male individuals and 17 (31.5%) were female individuals (Table 2). Among the 38 patients who were diagnosed with ACS, male individuals constituted a significant majority of 28 (73.7%) patients, compared to 10 (26.3%) female patients. The distribution was more balanced among 16 patients having non-cardiac chest pain, with nine (56.3%) male patients and seven (43.8%) female patients.

**Table 2:** Gender distribution of patients with ACS and non-cardiac chest pain. ACS, acute coronary syndrome; IPF, immature platelet fraction; MPV, mean platelet volume. Pearson’s correlation coefficient was applied to find the correlation of IPF with MPV and platelet count, and its statistical significance was checked by linear reg T test. A p-value of < 0.05 was considered statistically significant.

Gender	Number and percentage	Non-cardiac	ACS	Total	p-value
Female	No.	7	10	17	0.208
	%	43.8%	26.3%	31.5%	
Male	No.	9	28	37	
	%	56.3%	73.7%	68.5%	
Total	No.	16	38	54	
	%	100.0%	100.0%	100.0%	

**Figure 1:**
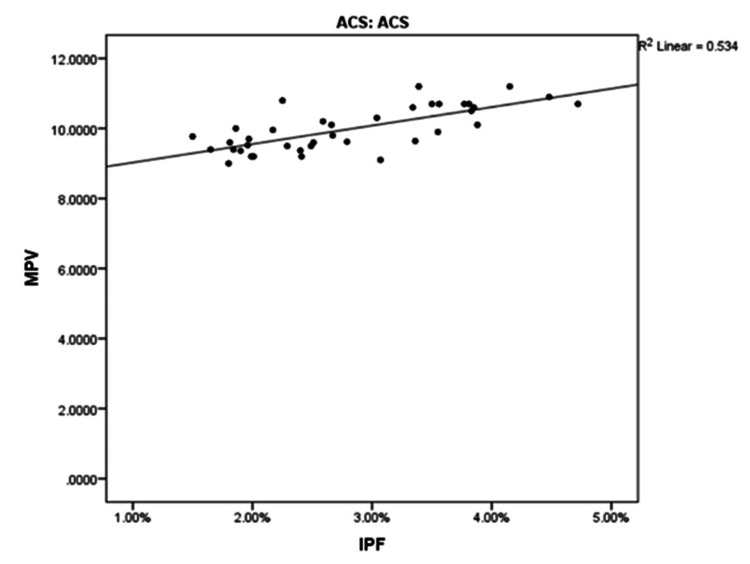
Correlation of IPF and MPV in ACS. ACS, acute coronary syndrome; IPF, immature platelet fraction; MPV, mean platelet volume.

Table 3 and Figure 1 display the correlation between IPF, MPV, and platelet count in patients with ACS and patients presenting with non-cardiac chest pain. In ACS patients, there is a high positive correlation between IPF and MPV (r = 0.731, p < 0.001), indicating that higher IPF levels are associated with higher MPV values. Conversely, MPV is negatively correlated with platelet count (r = - 0.311, p = 0.057) but is statistically not significant, suggesting that when MPV increases, platelet count decreases. This relationship highlights the potential role of elevated IPF and MPV as biomarkers in ACS, reflecting increased platelet activation, and in this condition.

**Table 3:** Correlation of IPF and MPV in ACS. ACS, acute coronary syndrome; IPF, immature platelet fraction; MPV, mean platelet volume. Pearson’s correlation coefficient was applied to find the correlation of IPF with MPV and platelet count, and its statistical significance was checked by linear reg T test. A p-value of < 0.05 was considered statistically significant.

Type of chest pain	Parameters	IPF	
		r-value	p-value
ACS	MPV	0.731	< 0.001
	Platelet count	- 0.311	0.057
Non-cardiac	MPV	0.069	0.801
	Platelet count	- 0.249	0.352

Table 4 depicts a comparative analysis of platelet parameters between patients with ACS and those with non-cardiac chest pain. The ACS group (38 patients) shows a significantly lower mean platelet count (234,789.47 ± 63,157.528) compared to the patients with non-cardiac chest pain (16 patients) with 309,875.00 ± 77,032.353 (p-value = < 0.001). Mean IPF is significantly higher in ACS patients (2.8111% ± 0.87219) compared to the patients with non-cardiac chest pain (1.3606% ± 0.26106) (p-value = < 0.001), indicating a greater turnover of platelets in ACS. MPV is also elevated in ACS patients (9.982632 ± 0.6295416) versus patients with non-cardiac chest pain (9.099375 ± 0.2777401) (p-value = < 0.001), suggesting larger and more reactive platelets in ACS. This difference shows statistical significance.

**Table 4:** Group statistics IPF and MPV. ACS, acute coronary syndrome; IPF, immature platelet fraction; MPV, mean platelet volume. Pearson’s correlation coefficient was applied to find the correlation of IPF with MPV and platelet count, and its statistical significance was checked by linear reg T test. A p-value of < 0.05 was considered statistically significant.

Parameters in ACS and non-cardiac chest pain		Frequency	Mean	Standard deviation	p-value
Platelet count	ACS	38	234789.47	63157.528	< 0.001
	Non-cardiac	16	309875.00	77032.353	
IPF	ACS	38	2.8111%	0.87219%	< 0.001
	Non-cardiac	16	1.3606%	0.26106%	
MPV	ACS	38	9.982632	0.6295416	< 0.001
	Non-cardiac	16	9.099375	0.2777401	

**Figure 2:**
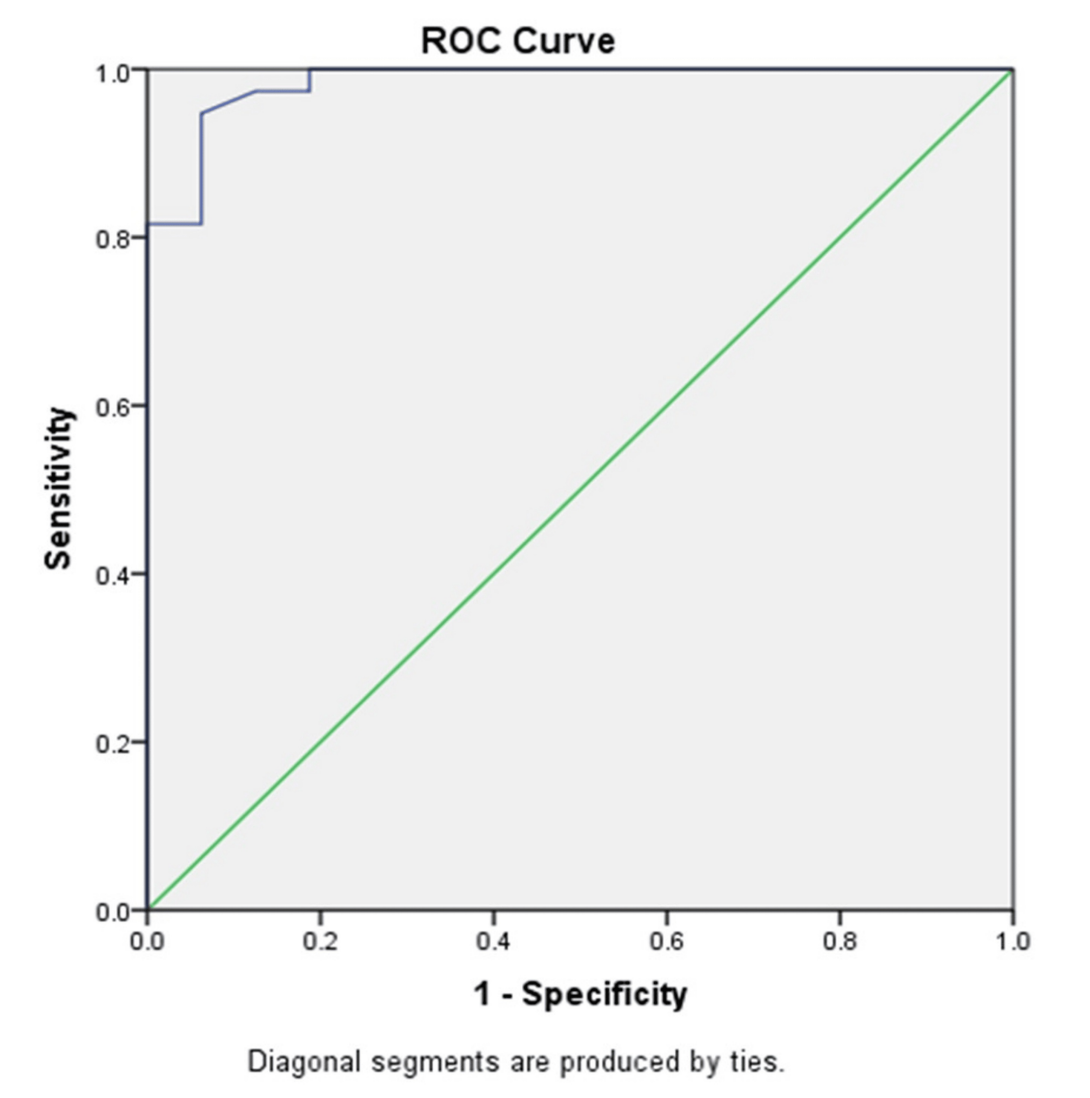
ROC (receiver operating characteristic) curve.

The ROC curve (Figure 2) and corresponding table (Table 5) assess the diagnostic accuracy of the IPF test. The area under the curve (AUC) is 0.984, with a standard error of 0.014, indicating adequate discrimination between positive and negative cases. The p-value was found to be significant (p < 0.001). So, IPF can be used as a biomarker for predicting ACS. The 95% confidence interval ranges from 0.958 to 1.000, reinforcing the reliability of the test. An optimal cut-off value for IPF in ACS appears to be greater than 1.7250%, where sensitivity is 94.7% and specificity of 93.7%. This suggests that a cutoff greater than this value (1.7250%) maximizes the IPF test's ability to correctly identify true positives while minimizing false positives, providing an effective diagnostic threshold for ACS.

**Table 5:** Area under the curve (AUC). a. Under the nonparametric assumption, b. Null hypothesis: true area = 0.5 Pearson’s correlation coefficient was applied to find the correlation of IPF (immature platelet fraction) with MPV (mean platelet volume) and platelet count, and its statistical significance was checked by linear reg T test. A p-value of < 0.05 was considered statistically significant.

Test result variable(s): IPF				
Area under the curve (AUC)	Standard error^a^	p-value^b^	95% confidence interval of AUC	
			Lower bound	Upper bound
0.984	.014	< 0.001	0.958	1.000

The data as per Table 6 suggests that IPF is not a strong differentiator between SVD, DVD, and TVD. DVD, with a single observation, has a mean IPF value of 3.34%, making it hard to generalize. SVD and TVD have similar mean IPFs (2.95% and 3.09%, respectively) with overlapping 95% confidence intervals, indicating no statistically significant difference. The overall IPF mean is 3.05%, with a standard deviation of 0.85850%, suggesting moderate variability. Therefore, based on this data, IPF alone does not provide a clear distinction between SVD, DVD, and TVD.

**Table 6:** Statistics of SVD, DVD and TVD with respect to IPF. ACS, acute coronary syndrome; IPF, immature platelet fraction; MPV, mean platelet volume; SVD, single vessel disease; DVD, double vessel disease; TVD, triple vessel disease. Pearson’s correlation coefficient was applied to find the correlation of IPF with MPV and platelet count, and its statistical significance was checked by linear reg T test. A p-value of < 0.05 was considered statistically significant.

Type of ACS	Frequency	Mean	Standard deviation	p-value
DVD	1	3.3400%		
SVD	5	2.9500%	1.01294%	0.925
TVD	5	3.0920%	.88361%	
Total	11	3.0500%	.85850%	

## Discussion

Our study aimed to determine the significance of IPF as a biomarker in the diagnosis of ACS. IPF and MPV were estimated in patients presenting with chest pain. Patients were followed up with ECG reports and cardiac enzyme (CK-MB {Creatine Kinase-MB} and HS Trop T {High-Sensitivity Troponin T}) reports, and ACS/non-cardiac chest pain was diagnosed and classified based on the data. STEMI patients, in whom CAG was performed, were further followed up to classify them as SVD, DVD, and TVD.Fifty-four patients with chest pain, including patients who presented to the ER and who got admitted in the ward and critical care unit (CCU), satisfying the inclusion criteria, were included in the study. ECG and cardiac enzymes were analyzed, and the patients were classified to be ACS and those having non-cardiac chest pain.

Among the 38 ACS patients, 11 (20.4%) patients were diagnosed with STEMI, 25 (46.3%) patients were diagnosed NSTEMI, and two (3.7%) patients were diagnosed with UA. IPF values were analyzed for each patient, the ROC curve was plotted, and the cut-off value of IPF for the diagnosis of ACS was found to be 1.7250%, with a sensitivity of 94.7% and a specificity of 93.7%. The AUC was found to be 0.984, with a standard error of 0.014 (p-value < 0.001).

Pearson’s correlation coefficient was applied to find the correlation of IPF with MPV and platelet count, and its statistical significance was checked by linear reg T test. In ACS patients, we found a strong positive correlation between IPF and MPV (r = 0.731, p < 0.001), indicating that higher IPF levels are associated with higher MPV values. Conversely, MPV is negatively correlated with platelet count (r = - 0.311, p < 0.001), suggesting that when MPV increases, platelet count decreases. This relationship observed in our study highlighted the potential role of elevated IPF and MPV as biomarkers in ACS, reflecting increased platelet activation and turnover in this condition.

In our study, the ACS group (38 patients) shows a significantly lower mean platelet count (234,789.47 ± 63,157.528) compared to the patients with non-cardiac chest pain (16 patients) with 309,875.00 ± 77,032.353 (p-value = < 0.001). Mean IPF is significantly higher in ACS patients (2.8111% ± 0.87219) compared to the patients with non-cardiac chest pain (1.3606% ± 0.26106) (p-value = < 0.001), indicating a greater turnover of platelets in ACS. MPV is also elevated in ACS patients (9.982632 ± 0.6295416) versus patients with non-cardiac chest pain (9.099375 ± 0.2777401) (p-value = < 0.001), suggesting larger and more reactive platelets in ACS. This difference shows statistical significance.

Behl et al. conducted a study examining the IPF, MPV, and interleukin-6 (IL-6) levels in ACS patients [15]. They found out that STEMI, NSTEMI, and UA showed a positive correlation between IPF and MPV, with correlation coefficients of 0.297, 0.531, and 1, respectively. Our study was in concordance with Behl et al., who, in their study, also concluded that elevated levels of IPF and MPV biomarkers can potentially aid in the early diagnosis of ACS [15].

Huang et al. investigated the clinical relevance of MPV and IPF in ACS patients [16]. Their study found that both MPV and IPF levels were significantly higher in ACS patients compared to controls, indicating their potential as biomarkers for early diagnosis of ACS and assessment of disease severity. These findings suggest that MPV and IPF can provide critical insights into platelet activation and inflammatory processes in ACS, which is also in favour of our study.

Further in our study, we used a T-test to differentiate SVD, DVD, and TVD using IPF with respect to CAG. DVD, with a single observation, has a mean IPF value of 3.34%, making it hard to generalize. SVD, with five samples, has a mean IPF of 2.95%, a standard deviation of 1.01294%, and a standard error of 0.45300%. The 95% confidence interval for the SVD group's mean ranges from 1.6923% to 4.2077%. Similarly, the TVD, also with five samples, exhibits a mean IPF of 3.092%, a standard deviation of 0.88361%, and a standard error of 0.39516%, with a 95% confidence interval from 1.9949% to 4.1891%. SVD and TVD have similar mean IPFs (2.95% and 3.09%, respectively) with overlapping 95% confidence intervals, indicating no statistically significant difference. The overall IPF mean is 3.05%, with a standard deviation of 0.85850% and a standard error of 0.25885% (with a 95% confidence interval ranging from 2.4733% to 3.6267%), suggesting moderate variability. Therefore, based on this data, IPF alone does not provide a clear distinction between SVD, DVD, and TVD.

Limitations

Our study included only 54 patients, which is a relatively small sample size. This limitation may affect the generalizability of our findings. Larger sample sizes are necessary to confirm the diagnostic value of IPF and MPV in diverse populations. The study was conducted in a single medical centre, which may limit the external validity of the results. Multicentric studies are required to validate our findings across different geographic locations and healthcare settings.

We attempted to determine the role of IPF in differentiating SVD, DVD, and TVD. Based on our data, IPF alone does not provide a clear distinction between SVD, DVD, and TVD due to the limited number of samples. In the future, studies with a more multicentric approach and with larger sample sizes might be able to demonstrate the role of IPF in differentiating SVD, DVD, and TVD.

## Conclusions

In our study, there was a strong positive correlation between IPF and MPV (r = 0.731, p < 0.001) in ACS patients. This indicates that elevated IPF levels are associated with higher MPV values, reflecting increased platelet activation and turnover, which are characteristic of ACS. Conversely, MPV is negatively correlated with platelet count (r = - 0.311, p < 0.001). DVD, with a single observation, has a mean IPF value of 3.34%, making it hard to generalize. SVD and TVD have similar mean IPFs (2.95% and 3.09%, respectively) with overlapping 95% confidence intervals, indicating no statistically significant difference.

In conclusion, to the best of our knowledge and data obtained, our study demonstrates that IPF and MPV are valuable biomarkers for the diagnosis of ACS. Future research should focus on larger, multi-centric studies to validate our findings and further explore the clinical applications of IPF and MPV in ACS diagnosis and prognosis.
